# Training Deep Spiking Convolutional Neural Networks With STDP-Based Unsupervised Pre-training Followed by Supervised Fine-Tuning

**DOI:** 10.3389/fnins.2018.00435

**Published:** 2018-08-03

**Authors:** Chankyu Lee, Priyadarshini Panda, Gopalakrishnan Srinivasan, Kaushik Roy

**Affiliations:** Nanoelectronics Research Laboratory, School of Electrical and Computer Engineering, Purdue University, West Lafayette, IN, United States

**Keywords:** spiking neural network, convolutional neural network, spike-based learning rule, spike timing dependent plasticity, gradient descent backpropagation, leaky integrate and fire neuron

## Abstract

Spiking Neural Networks (SNNs) are fast becoming a promising candidate for brain-inspired neuromorphic computing because of their inherent power efficiency and impressive inference accuracy across several cognitive tasks such as image classification and speech recognition. The recent efforts in SNNs have been focused on implementing deeper networks with multiple hidden layers to incorporate exponentially more difficult functional representations. In this paper, we propose a pre-training scheme using biologically plausible unsupervised learning, namely Spike-Timing-Dependent-Plasticity (STDP), in order to better initialize the parameters in multi-layer systems prior to supervised optimization. The multi-layer SNN is comprised of alternating convolutional and pooling layers followed by fully-connected layers, which are populated with leaky integrate-and-fire spiking neurons. We train the deep SNNs in two phases wherein, first, convolutional kernels are pre-trained in a layer-wise manner with unsupervised learning followed by fine-tuning the synaptic weights with spike-based supervised gradient descent backpropagation. Our experiments on digit recognition demonstrate that the STDP-based pre-training with gradient-based optimization provides improved robustness, faster (~2.5 ×) training time and better generalization compared with purely gradient-based training without pre-training.

## 1. Introduction

In this era of data deluge with real-time content continuously generated by distributed sensors, intelligent neuromorphic systems are required to efficiently deal with the massive amount of data and computations in ubiquitous automobiles and portable edge devices. Spiking Neural Networks (SNNs), often regarded as third generation brain-inspired neural networks (Maass, 1997), can be highly power-efficient and have competitive capabilities to deal with several cognitive tasks (Khan et al., 2008; Jo et al., 2010; Merolla et al., 2014). A spiking neuron, one of the core building blocks of SNNs, transmits information in the form of electric event pulses (or spikes) through plastic synapses. Event-driven computing capability is a fundamental property of SNNs that enables sparse and irregular input encoding, leading to low latency and power consumption. Till now, two-layer (shallow) fully-connected SNN architectures have been widely explored for classification and recognition tasks (Brader et al., 2007; Diehl and Cook, 2015; Zhao et al., 2015). However, they necessitate large number of trainable parameters to attain competitive classification accuracy, which constrains their scalability for complex applications. Recent developments on multi-layer SNNs, composed of an input layer followed by two or more hidden layers and an output layer, address this scalability issue (Kheradpisheh et al., 2016; Lee et al., 2016; O'Connor and Welling, 2016; Panda and Roy, 2016). Multi-layer neural networks allow the systems to hierarchically classify the complex input patterns by building feature hierarchies. The early layer detects elementary representations of input patterns while the subsequent layers capture the higher-level concepts comprising elementary features. Nevertheless, the training of deep SNNs remains an intricate and challenging problem.

Training strategy for SNNs can be broadly categorized into unsupervised and supervised algorithms. Unsupervised algorithms discover the characteristics and underlying structures of input patterns without using the corresponding output labels. Spike-Timing-Dependent-Plasticity (STDP) (Bliss and Collingridge, 1993; Bi and Poo, 1998; Song et al., 2000) is a bio-plausible unsupervised learning mechanism that instantaneously manipulates the synaptic weights based on the temporal correlations between pre- and post-synaptic spike timings. It is a simple and fast training method, which accounts for the history of pre- and post-synaptic spikes between two adjacent (local) layers. However, the resultant classification accuracy with STDP training alone is still lower than the state-of-the-art results (Wan et al., 2013; He et al., 2016). On the other hand, supervised learning extracts internal representation given the training examples and target output labels. The standard gradient descent error backpropagation (BP) algorithm (Rumelhart et al., 1985), which is typically used for achieving the state-of-the-art classification performance in a frame-based deep learning, modifies the network parameters in order to minimize the designated output loss, which is a function of the difference between the predicted and desired outputs. In the context of SNNs, supervised learning has been utilized to train the network off-line with continuous input signals as in an Artificial Neural Network (ANN) and substitute the artificial neurons with spiking neurons for efficient inference (Cao et al., 2015; Hunsberger and Eliasmith, 2015; Diehl et al., 2016; Rueckauer et al., 2017; Sengupta et al., 2018). However, the possibility of incurring accuracy loss during the conversion from ANN to SNN together with highly efficient event-driven computing capability of SNNs have motivated recent works that directly train SNNs using BP algorithm through input spike events (Lee et al., 2016; Panda and Roy, 2016; Mostafa, 2017; Neftci et al., 2017; Stromatias et al., 2017). The spike-based BP introduced in Panda and Roy (2016) treats the membrane potential as a differentiable activation of the spiking neuron to layer-wise train the weights using a spike-based auto-encoder scheme. Lee et al. (2016) has taken forward spike-based BP to calculate final loss and back-propagate error for end-to-end supervised gradient descent optimization. The spike-based BP is one successful method for training deep SNNs, but there are several challenges. First, it is compute-intensive and requires a large amount of data and effort, which impedes the networks from accomplishing efficient on-chip learning on cognitive tasks. The procedures for computing the derivative of loss function with respect to the parameters are complicated and necessitates lots of training examples to generalize well to previously unseen data while avoiding overfitting on training examples. Second, training neural networks comprising many non-linear layers is a problematic multi-dimensional non-convex optimization problem that does not have a distinct global minima. Therefore, it is hard to find an optimal initial condition of the synaptic weights and the neuronal thresholds, which are required to deal with chaotic convergence behavior and facilitate stable training convergence. To overcome these impediments, appropriate network initialization and optimization/regularization tools are essential for training deep SNNs.

Given the deep hierarchical SNN models, it is still unclear which learning algorithm (i.e., unsupervised or supervised) is suitable for training the systems. Both the STDP and spike-based BP learning have been demonstrated to capture hierarchical features in SNNs (Masquelier and Thorpe, 2007; Kheradpisheh et al., 2016; Lee et al., 2016, 2018; O'Connor and Welling, 2016; Panda and Roy, 2016; Panda et al., 2017), but the insufficient classification performance of standalone STDP-trained networks, overfitting issues and unstable convergence behaviors of BP algorithm are a couple of obstacles toward efficient learning. To that effect, we propose leveraging STDP-based unsupervised learning that encourages the hidden layers to discover useful characteristics and structures of input patterns prior to the gradient-based supervised optimization. In this work, the multi-layer convolutional neural networks comprising of the convolutional and pooling layers followed by successive fully-connected layers are populated with bio-plausible leaky integrate-and-fire spiking neurons (Dayan and Abbott, 2001) to deal with sparse Poisson-distributed spike trains that encodes the pixel intensity in its firing rate. The proposed pre-training scheme trains the convolutional kernels using STDP algorithm in a layer-wise manner that enables them to self-learn features from input spike patterns. The pre-training enforces inductive bias to network parameters including the synaptic weights and neuronal thresholds, which provides a better starting point compared to random initialization. After finishing the pre-training, gradient descent BP algorithm fine-tunes the synaptic weights across all the layers leading toward the optimum local minima. The proposed strategy of using both the unsupervised and supervised learning algorithm can be referred to as “semi-supervised learning.” We believe that biologically plausible unsupervised learning and state-of-the-art supervised deep learning algorithms can pave ways to jointly optimize the hierarchical SNNs for achieving efficient and competitive performance at the level of human brain.

The rest of the paper is organized as follows. In section 2, we explain the fundamentals and architecture of deep convolutional SNNs. Next, we describe the proposed semi-supervised training methodology, which includes the STDP-based unsupervised pre-training and BP-based supervised fine-tuning algorithms. In section 3, we present the simulation results, which validate the efficacy of the semi-supervised training methodology for MNIST handwritten digit recognition. In section 4, we discuss the contributions of the proposed method and investigate how the pre-training helps the gradient-based optimization procedure. Finally, we conclude the paper in section 5.

## 2. Materials and methods

### 2.1. SNN fundamentals and network architecture

#### 2.1.1. Computational models of spiking neurons and synapses

We use the biologically plausible Leaky-Integrate-and-Fire (LIF) model (Dayan and Abbott, 2001) for simulating the dynamics of a spiking (post) neuron that is driven by the input (pre) neurons via plastic synapses. The LIF neuron integrates the input spikes modulated by the inter-connecting synaptic weights, leading to a change in its membrane potential (*V*_*mem*_) whose temporal dynamics are formulated below.

(1) $$\begin{array}{r} \tau_{m}\frac{dV_{mem}}{dt}=-V_{mem}+w*\theta \left(t-t_{k}\right), \end{array}$$

The incoming spike (Dirac-delta pulse) occurring at time instant *t*_*k*_, denoted by θ (*t*−*t*_*k*_), gets modulated by the synaptic weight (*w*) to produce resultant current that is integrated by the post-neuron in its membrane potential. The membrane potential leaks exponentially subsequent to the removal of the input spike. The time constant, τ_*m*_, determines the rate of membrane leakage over time, where a smaller value incurs a faster membrane potential decay and vice versa. When the accumulated membrane potential reaches a certain firing threshold, the LIF neuron fires an output spike to the fan-out synapses and is thereafter reset. The non-linear membrane potential decay and reset mechanisms help regulate the spiking activities of the post-neurons.

#### 2.1.2. Multi-layer convolutional spiking neural network topology

**Figure 1:**
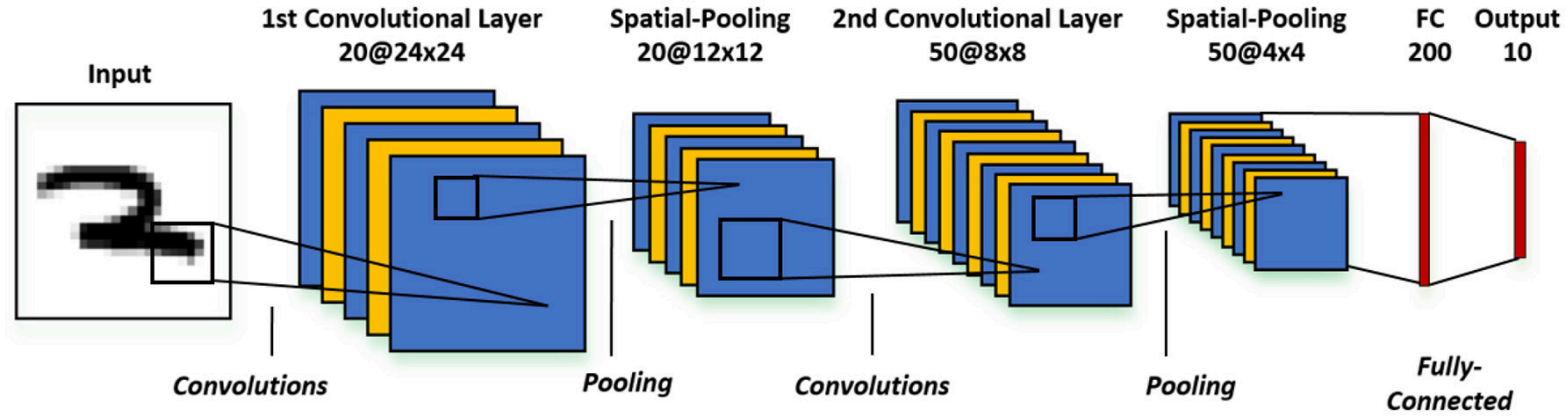
Architecture of the multi-layer convolutional spiking neural network consisting of an input layer, alternating convolutional and spatial-pooling layers, and final fully-connected layers for inference.

The recognition of high-dimensional input patterns necessitates multi-layer network topologies that can effectively learn hierarchical representations from input stimuli. In this work, we use a convolutional neural network model that consists of an input layer followed by intermediate hidden layers and the final output layer as illustrated in Figure 1. The input layer encodes the images as Poisson-distributed spike trains where the probability of spike generation is proportional to the pixel intensity. The hidden layers composed of alternating convolutional (C) and spatial-pooling (P) layers represent the intermediate stages of feature hierarchies. The spikes from the hidden layers are combined sequentially for final classification by the fully-connected (FC) layers. The convolutional and fully-connected layers consist of trainable parameters while the spatial-pooling layers are fixed a priori. The weight kernels constituting the convolutional layers encode the feature representations at multiple hierarchical levels. The adapted convolutional kernels can appropriately detect the spatially correlated local features in the input patterns as a result of convolution, which inherently renders the network invariant to translation (shift) in the object location. Next, the spatial-pooling layer downscales the dimension of the feature maps produced by the previous convolutional layer while retaining the spatial correlation between neighborhood pixels in every feature map. For instance, a fixed 2 × 2 kernel (each having a weight of 0.25) strides through a convolutional feature map without overlapping and fires an output spike at the corresponding location in the pooled feature map if the summed spikes of the 4 input pixels within the window exceeds a threshold of 0.8. The pooling operation offers the following key benefits. First, it provides small amount of additional network invariance to input transformations while reducing the dimension of the convolutional feature maps. Furthermore, the pooling operation, by the virtue of downscaling the feature maps, enlarges the effective size of convolutional kernels in the subsequent layer. This helps successive convolutional layers to efficiently learn hierarchical representations from low to high levels of abstractions. The number of pooled feature maps is equal to the number of convolutional feature maps. The feature maps of the final pooling layer are unrolled into a 1−*D* vector that is fully-connected to the output layer which produces inference decisions. The fully-connected layer acts as a classifier to effectively incorporate the composition of features resulting from the alternating convolutional and pooling layers into the final output classes.

### 2.2. Proposed semi-supervised learning methodology

The proposed semi-supervised learning methodology is comprised of unsupervised pre-training followed by supervised fine-tuning using a spike-based gradient descent BP algorithm in a global fashion. The concept of unsupervised pre-training was introduced in Hinton et al. (2006) to efficiently train artificial deep belief nets, a generative model comprising several stacked restricted Boltzmann machines, using a fast greedy layer-wise training algorithm. In Bengio et al. (2007), Erhan et al. (2009), and Vincent et al. (2010), the authors employed unsupervised learning mechanisms such as contrastive divergence and de-noising auto-encoder to hierarchically pre-train successive layers of deep belief nets. In spiking domain, Kheradpisheh et al. (2016); Panda and Roy (2016); Tavanaei and Maida (2016, 2017); Ferré et al. (2018); Lee et al. (2018) have explored semi-supervised learning to train deep SNNs, with layer-wise unsupervised learning using spike-based auto-encoder/sparse-coding/STDP-based methods followed by supervised learning at the final classification layer. However, we use STDP-based unsupervised pre-training to discover useful characteristics and underlying structures of data to appropriately condition and initialize the synaptic weights and neuronal firing thresholds for a given pattern recognition task. After pre-training the network, we use the spike-based gradient descent BP algorithm to fine-tune the synaptic weights end-to-end in a manner that minimizes discrepancy between the actual outputs and target labels. We now describe the individual STDP-based unsupervised and BP-based supervised learning mechanisms.

#### 2.2.1. Unsupervised pre-training using spike-timing-dependent-plasticity

**Figure 2:**
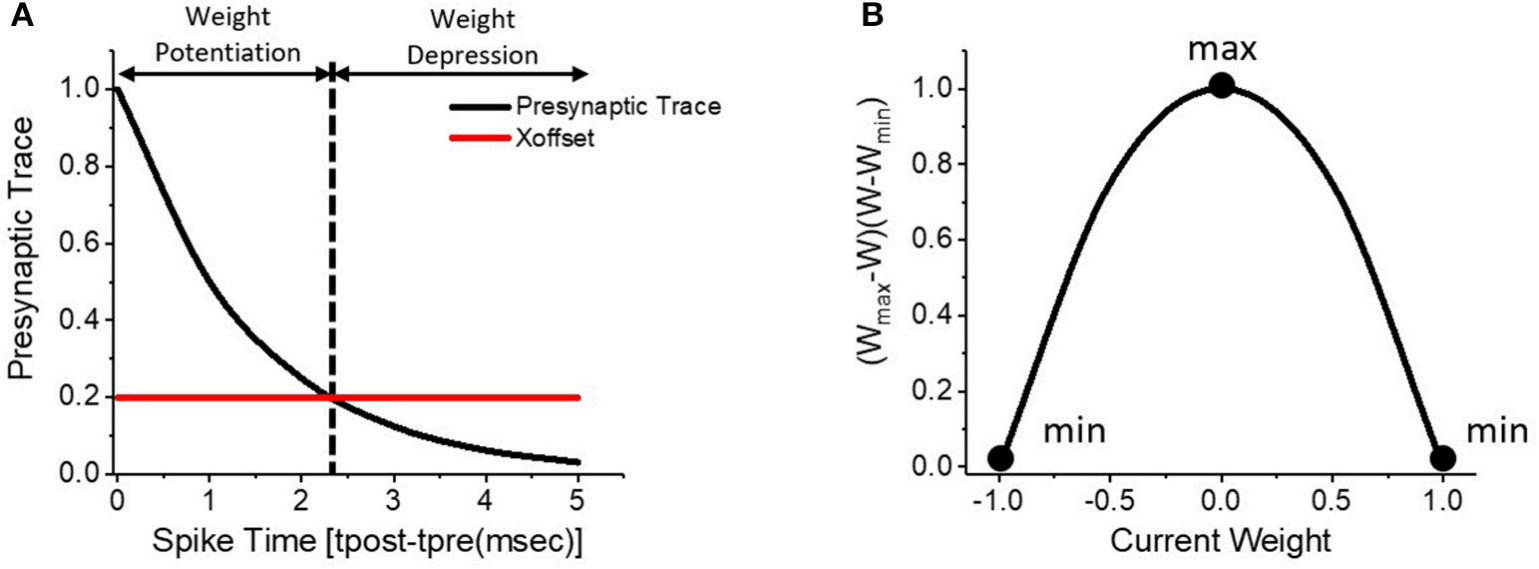
**(A)** Weight-dependent positive-STDP learning rule, where the synaptic weight is potentiated for strong timing correlation between the pre- and post-synaptic spikes and depressed for larger spike timing differences. **(B)** Illustration of the non-linear weight-dependent updates to the synaptic weight.

Spike-Timing-Dependent-Plasticity (STDP) is a biologically plausible unsupervised learning mechanism that self-learns synaptic weights based on the degree of temporal correlations between the pre- and post-synaptic spike events. As shown in Figure 2A, the pre-synaptic trace resets to 1 when pre-synaptic spike arrives and exponentially decays over time. Hence, the pre-synaptic trace encodes the timing correlation between pre- and post-neuronal spikes in the positive timing window. The strength (weight) of synapse is potentiated if a pre-synaptic spike triggers the post-neuron within a period of time that is determined by a threshold, namely χ_*offset*_. The synaptic weight is depressed for larger spike timing differences. The STDP weight updates are applied to the synapses only at the time instances of post-synaptic firing. Specifically, we use the weight-dependent positive-STDP rule whose characteristic is formulated as follows.

(2) $$\begin{array}{r} \Delta w=\eta_{STDP}\left(e^{\frac{t_{pre}-t_{post}}{\tau_{pre}}}-\chi_{offset}\right)\left(w_{max}-w\right)\left(w-w_{min}\right) \end{array}$$

where Δ*w* is the change in the synaptic weight, η_*STDP*_ is the learning rate, *t*_*pre*_–*t*_*post*_ is the timing difference between pre- and post-synaptic spikes, τ_*pre*_ is the time constant controlling the length of the STDP timing window, and *w*_*max*_ (*w*_*min*_) is the maximum (minimum) bound on the synaptic weight. The amount of weight change has a non-linear dependence on the current weight (*w*), which is specified by the product of (*w*_*max*_-*w*) and (*w*-*w*_*min*_). Smaller the absolute value of the current weight, larger is the ensuing weight change and vice versa as illustrated in Figure 2B. Such nonlinear weight-dependent updates ensure a gradual increase (decrease) of the synaptic weight toward the maximum (minimum) bound, thereby improving the efficiency of synaptic feature learning. Note that the synaptic weights are locally updated in an unsupervised way based on the spiking behaviors of pre-/post-neurons at adjacent layers.

In convolutional SNNs, the weight kernels locally inter-connecting the successive layers stride over the pre-neuronal maps to construct the output feature maps at every time step. In an event of a post-spike, the time difference between corresponding pre- and post-neuronal spikes is used to conduct individual STDP update on the convolutional weights. In case of multiple post-neuronal spikes in an output feature map, averaged STDP updates are applied to the kernel weights. Accordingly, the STDP learning enables the weight kernels to self-learn useful features from the complex input patterns. In addition to performing STDP updates on the weight kernels, we modulate the firing threshold of the units in the corresponding feature map to enable kernels (among the feature maps in a convolutional layer) to learn different representations of input patterns. In the event of a post-neuronal spike in a convolutional feature map, we uniformly increase the firing threshold of all the post-units constituting the feature map. In the period of non-firing, the firing threshold of the feature map exponentially decays over time. Such threshold adaptation, referred as homeostasis (Clopath et al., 2010), balances the firing threshold with respect to the strength of kernel weights and effectively prevents convolutional kernels in a feature map from dominating the learning. In addition, the negative synaptic weights preclude the need for lateral inhibitory synaptic connections among feature maps in a layer (by regulating spiking activities of units within feature map) that is otherwise essential for competitive feature learning. In previous studies, STDP learning has been demonstrated to self-learn convolutional kernels layer-by-layer for training multi-layer convolutional SNNs (Kheradpisheh et al., 2016; Lee et al., 2018). In this work, we exploit the unsupervised feature learning capabilities of STDP learning for appropriately initializing the convolutional weights and corresponding neuronal firing thresholds in multi-layer systems. We greedily pre-train each convolutional layer one at a time using the unsupervised STDP learning and uniform threshold adaptation scheme. We begin by training the first convolutional layer that enables the weight kernels to discover low-level characteristic features from input patterns in an unsupervised manner. At every time step, the convolutional kernels slide over the input maps to detect the characteristic features and construct output feature maps. The unit in output feature maps fires when the convolutional kernel captures the characteristic features, and the weight kernel is updated with STDP and the threshold adaptation mechanism. After the first convolutional layer is trained, the adjusted weight kernels and neuronal firing thresholds are frozen to feed the input again for estimating the average firing rate of units in the output feature maps. The generated feature maps of first convolutional layer (the nonlinear transformations of inputs) are spatially pooled and passed to the next convolutional layer to extract the higher-level representations in hierarchical models. This process is repeated until all convolutional layers are pre-trained. Note that we do not modify the synaptic weights of the fully-connected layer (or the classifier) during the pre-training procedure. Therefore, the unsupervised pre-training mechanism, in essence, initially finds out unique features and underlying structures of input patterns for the task at hand prior to supervised fine-tuning.

#### 2.2.2. Supervised fine-tuning using spike-based backpropagation

In this sub-section, we first discuss the standard supervised backpropagation (BP) learning that is a widely used first-order gradient descent algorithm for ANN (Rumelhart et al., 1985), and subsequently detail its spike-based adaptation used in this work. The standard BP algorithm involves forward propagation and error back-propagation. During the forward propagation, an input pattern and its output (target) label are respectively presented to measure the corresponding loss function, which is a function of discrepancy between target labels and predicted outputs from the current network parameters. The error backpropagation is thereafter used to compute the gradients of the loss function with respect to each synaptic weight for determining their contributions to the final output loss. The synaptic weights are modified based on the individual gradient in the direction to minimize the output loss. The above steps are iteratively applied over mini-batches of input patterns to obtain the optimal network parameters, which facilitate the training loss to converge to a local minima. In this work, the standard BP technique is adapted for SNNs by taking into account the event-driven characteristics for optimizing the weights directly using the spike input signals. It is important to note that the primary difference between ANNs and SNNs lies in the dynamics of the output produced by the respective neuron models. The spiking neurons communicate over time by means of spike pulses that are discrete and non-differentiable signals. This is in stark contrast with the differentiable continuous (scalar) values from the artificial neurons such as *sigmoid*, *tanh*, and *ReLU* functions (Krizhevsky et al., 2012; Goodfellow et al., 2016). In spike-based BP algorithm, we low-pass filtered the post-spike trains to obtain a pseudo derivative by creating differentiable activation function (explained below). This allows the final output loss to be propagated backward to hidden layers for updating the associated synaptic weights suitably.

**Figure 3:**
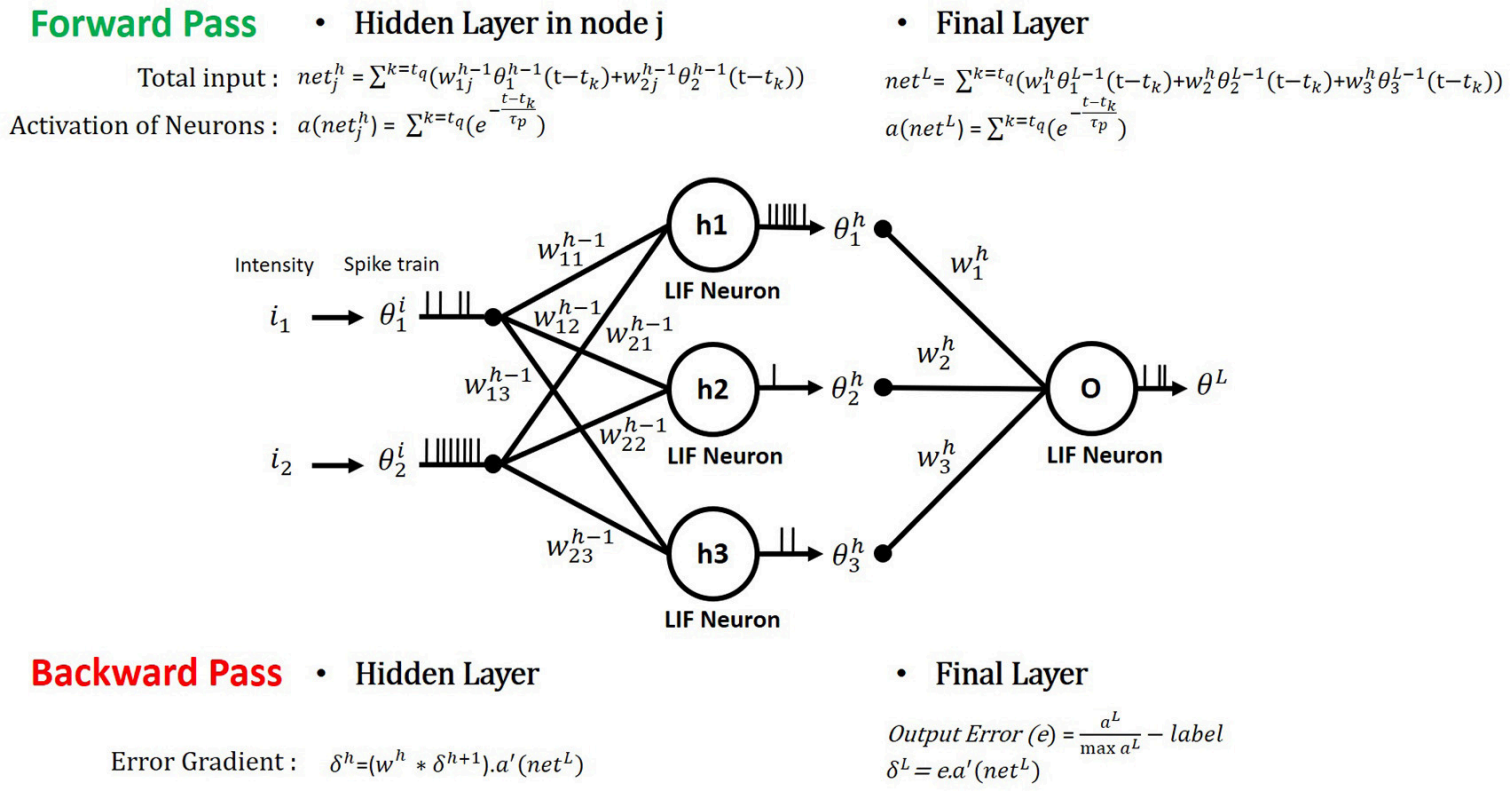
Illustration of spike forward and backward propagation of a multi-layer SNN consisting of LIF neurons. In forward pass, the spiking neuron integrates the input current (net) generated by the weighted sum of the pre-neuronal spikes with the interconnecting synaptic weights and produces an output spike train. In backward pass, the derivatives of designated loss function with respect to each synaptic weight are calculated from chain-rule.

During the forward propagation, the input pixel values are converted to Poisson-distributed spike trains and directly fed to the SNN. The sum of Dirac-delta pulses (denoted by *x*_*i*_ for the *i*_*th*_ input neuron) are weighted by inter-connecting synaptic weights ($w_{ij}^{l}$) to be integrated to post-neurons as illustrated in Figure 3 and formulated as (3).

(3) $$\begin{array}{r} net_{j}^{l+1}\left(t\right)=\overset{n^{l}}{\underset{i=1}{\sum }}w_{ij}^{l}*x_{i}\left(t\right),wherex_{i}\left(t\right)=\overset{t}{\underset{k=1}{\sum }}\theta_{i}\left(t-t_{k}\right) \end{array}$$

where ${net}_{j}^{l+1}$ is the resultant current received by the *j*_*th*_ post-neuron at (*l*+1)_*th*_ layer, *n*^*l*^ denotes the number of neurons in *l*_*th*_ layer, *t*_*k*_ represents the time instant at which pre-neuron spikes. The post-neurons fire output spikes when the respective membrane potentials exceed a definite neuronal firing threshold, after which the potentials are reset and the spikes are broadcast to the subsequent layer. This process is successively carried out by the post-neurons in every layer based on the incoming spikes received from the preceding layer to produce spike trains over time as shown in Figure 3. The “differentiable activation” of the spiking neuron, which defines the highly non-linear relationship between the weighted pre-neuronal spikes and post-spike firing rate, is generated by low-pass filtering the individual post-spike train as formulated below.

(4) $$\begin{array}{rcl} Activation\;of\;neuron,a_{j}\left(t\right) & = & \overset{t}{\underset{k=1}{\sum }}exp\left(-\frac{t-t_{k}}{\tau_{p}}\right) \end{array}$$

(5) $$\begin{array}{rcl} Final\;output\;error,e_{j} & = & \frac{a_{j}^{L}}{max\left(a^{L}\right)}-label_{j} \end{array}$$

(6) $$\begin{array}{rcl} Loss\;function,E & = & \frac{1}{2}\overset{n^{L}}{\underset{j=1}{\sum }}e_{j}^{2} \end{array}$$

The activation, *a*_*j*_, of an LIF neuron is computed by integrating the unit spikes [at time instants *t*_*k*_ in (4)] and decaying the resultant sum in the time period between successive spikes. The time constant (τ_*p*_), which determines the rate of decay of the neuronal activation, accounts for the non-linear membrane potential decay and reset mechanisms that influence the spiking dynamics of the post-neuron. The activation of the output neurons in the fully-connected layer (classifier) is normalized to obtain a probability distribution over all final class predictions for a given input pattern. The final error (*e*_*j*_) for each output neuron is evaluated by comparing the normalized output activation with the target label (*label*_*j*_) of the presented input as shown in (5). The corresponding loss function [*E* in (6)] is defined as the mean square of the final error over all the output neurons.

Next, we detail the gradient descent backpropagation algorithm that is used to minimize the output loss in SNNs. We first estimate the gradients of the output loss with maximum likelihood at the final output layer and back-propagate the gradients all the way down through the network using recursive chain rule (Rumelhart et al., 1985). The gradient with respect to the weights of hidden layers are obtained as described by the following equations.

(7) $$\begin{array}{rcl} \delta^{L} & = & e.a^{\prime }\left(net^{L}\right) \end{array}$$

(8) $$\begin{array}{rcl} a^{\prime }\left(net^{L}\right) & = & a^{\prime }\left(t\right)+1=\overset{t}{\underset{k=1}{\sum }}\left(-\frac{1}{\tau_{p}}e^{-\frac{t-t_{k}}{\tau_{p}}}\right)+1 \end{array}$$

(9) $$\begin{array}{rcl} \delta^{h} & = & \left({\left(w^{h}\right)}^{T}*\delta^{h+1}\right).a^{\prime }\left(net^{h}\right) \end{array}$$

The quantity, δ^*L*^, henceforth referred as the “error gradient,” represents the gradient of the output loss with respect to the net input current received by the post-neurons in the final output layer. It can readily be computed [as shown in (7)] by multiplying the final output error [e in (5)] with the derivative of the corresponding post-neuronal activation (*a*′(*net*^*L*^)) in (8). Note that “.” denotes element-wise multiplication while “*” indicates matrix multiplication in the Equations (3–10). The neuronal activation [as described in (4)] is non-differentiable with respect to input current because of discrete time series output signals. To overcome this, we obtain a pseudo-derivative of post-neuronal activation by adding a unity value to the time derivative of the corresponding activation as formulated in (8). The time derivative of neuronal activation reflects highly non-linear (leaky) characteristics of LIF neuron model and adding a unity value facilitates ignoring the discontinuity (step jump) that arises at each spike time. The error gradient, δ^*h*^, at any hidden layer is recursively estimated by back-propagating the error gradient from the successive layer [(^*w*^*h*^)T^*δ^*h*+1^] and multiplying it with the derivative of neuronal activation [(a′(*net*^*h*^)] as formulated in (9). It is worth mentioning here that the presented spike-based BP algorithm mitigates the vanishing gradient phenomena, because the derivatives of the spiking neuronal activation [shown in (8)] do not saturate unlike saturating activation functions.

(10) $$\begin{array}{rcl} △w^{l} & = & \frac{a^{l}}{max\left(a^{l}\right)}*{\left(\delta^{l+1}\right)}^{T} \end{array}$$

(11) $$\begin{array}{rcl} w^{l} & = & w^{l}-\eta_{BP}△w^{l} \end{array}$$

The derivative of the output loss with respect to the weights interconnecting the layers *l* and *l*+1 [△*w*^*l*^ in (10)] is determined by multiplying the transposed error gradient at *l*+1 (δ^*l*+1^) with the normalized activation of the neurons in layer *l*. In case of convolutional neural networks, we back-propagate the error in order to get the partial derivatives of the loss function with respect to the given output feature map. Then, we average the partial derivatives over the output map connections sharing the particular weight to account for the effective updates of kernel weights. Finally, the calculated partial derivatives of loss function are used to update the respective weights using a learning rate (η_*BP*_) as illustrated in (11). Iteratively updating the weights over mini-batches of input patterns leads the network state to a local minimum, thereby enabling the network to capture hierarchical internal representations of the data.

## 3. Results

In this section, we demonstrate the capability of the proposed semi-supervised learning strategy on the handwritten digit MNIST dataset (LeCun et al., 1998) using a MATLAB-based custom simulation framework. The MNIST dataset contains 60k training and 10k testing (grayscale) images belonging to 10 categories. For the experiments, we implement relatively shallow and deep multi-layer convolutional SNN topologies, which comprise of 28x28 input image, convolutional (C) layers using 5 × 5 sized weight kernels, spatial-pooling (P) layers with 2 × 2 non-overlapping pooling regions followed by successive fully-connected (FC) layers. The detailed multi-layer neural network topologies are as follows: the shallow network is 28 × 28−36*C*5−2*P*−10*FC* and the deep network is 28 × 28−20*C*5−2*P*−50*C*5−2*P*−200*FC*−10*FC*. The initial synaptic weights are randomly assigned at each layer following the weights initialization scheme (Lee et al., 2016). The neuronal firing thresholds (*v*_*th*_) are set proportional to the strength of the synaptic distribution as shown below.

(12) $$\begin{array}{r} w^{l}\in U\left[-\sqrt{\frac{3}{n^{l}}},\sqrt{\frac{3}{n^{l}}}\right],v_{th}=\alpha \sqrt{\frac{3}{n^{l}}},\alpha >0 \end{array}$$

where *w*^*l*^ denotes the synaptic weight matrix connecting layers *l* and *l*+1, *U*[−*k, k*] indicates the uniform distribution in the interval between *k* and *k*, and *n*^*l*^ is the size of the *l*_*th*_ layer.

We train the multi-layer convolutional SNNs using the proposed semi-supervised learning strategy, which comprises initial unsupervised pre-training and subsequent supervised fine-tuning (or spike-based BP) procedures using the parameters listed in Table 1. In every iteration of training, a subset (mini-batch) of randomly sampled training images are fed to the system such that the static inputs are converted stochastically into spike events, wherein the firing rate encodes the pixel intensity. During the unsupervised pre-training, we present a fraction of training data to the network for 25 ms (assuming a simulation time-step of 1 ms) and adjust each convolutional layer one at a time. After the layer-wise pre-training of convolutional layers, the kernel weights with respect to the neuronal firing threshold are appropriately initialized and conditioned for further fine-tuning. Next, we conduct gradient-based BP learning, which evaluates the gradients of a loss function with respect to the synaptic weights through forward and backward propagations. During supervised fine-tuning, we present all training samples (excluding the ones used for pre-training) for 100 ms in the first epoch and full-training samples for 50 ms in subsequent epochs. Note that passing the full training examples once through a network denotes an epoch, which consists of 600 iterations in case of MNIST dataset given the mini-batch size of 100. The learning rate is kept constant throughout the unsupervised and the supervised learning, respectively.

**Table 1:** Parameters used in the experiments.

**Parameter**	**Value**
STDP Type	Positive STDP
Decay Constant of Membrane Potential (τ_*m*_)	10 ms
Decay Constant of Synaptic Trace (τ_*pre*_)	1.5 ms
Decay Constant of Post-neuronal Activation Function (τ_*p*_)	100 ms
Training Time Duration (STDP, BP)	25, 100, 50 ms
Inference Time Duration	200 ms
Mini-batch Size	100
Maximum Input Rate (STDP, BP, Inference)	200, 500, 500 Hz
Convolutional Kernel Size/Stride	5 × 5, 1
Spatial-pooling Non-overlapping Region/Stride	2 × 2, 2
Threshold Initialization Constant (α) for,C, FC Layer without Pre-training	5, 3

**Figure 4:**
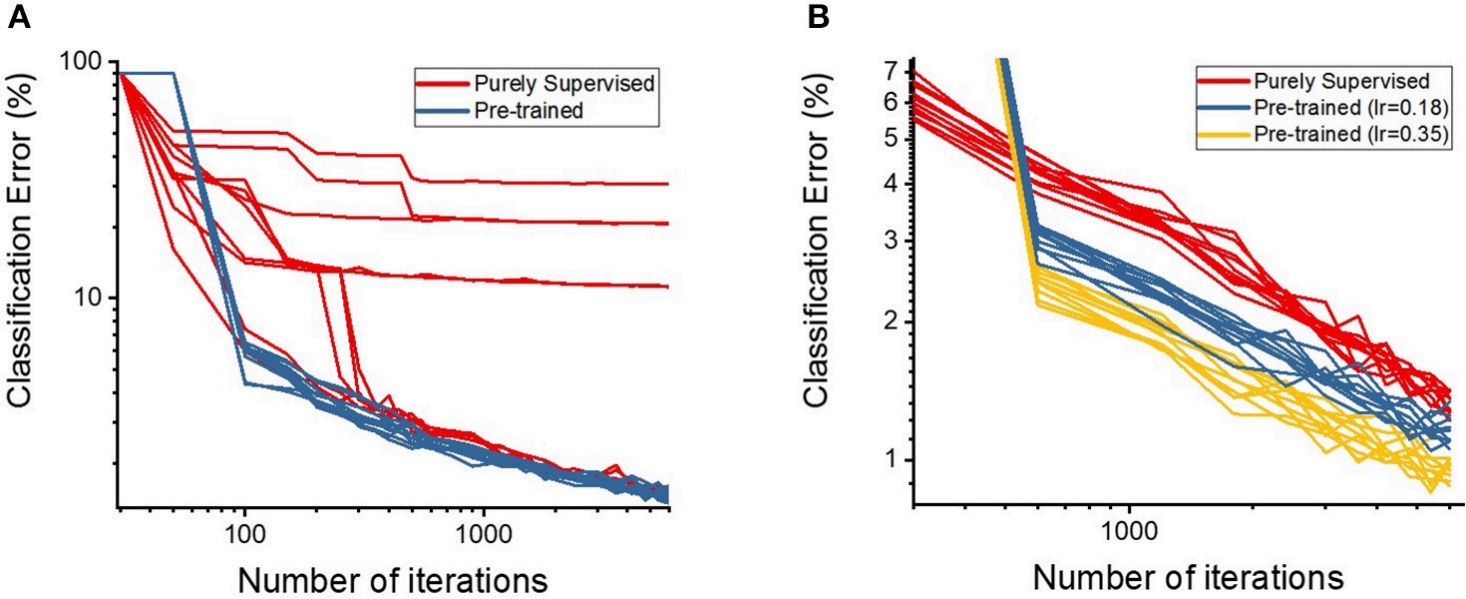
The classification accuracies (in log scale) on **(A)** shallow and **(B)** deep multi-layer convolutional spiking neural networks of pre-trained and supervised model starting from different states of randomly initialized synaptic weights.

First, we discuss the effectiveness of our semi-supervised learning methodology by evaluating the classification performance of the shallow and deep multi-layer networks on the MNIST test dataset. We compare our proposed semi-supervised training strategy (i.e., pre-trained model) against standalone gradient-based supervised optimization without pre-training (i.e., purely supervised model) for both shallow and deep networks. The spike-based gradient descent training follows an identical criterion in both pre-trained and purely supervised models with the exception of parameter initialization (i.e., unsupervised STDP-based pre-training vs. random initialization). Figure 4A shows the classification error comparison between the two scenarios for shallow multi-layer network, which started from 10 different initialization of the weight state. Note, the learning rate across the 10 different cases for both pre-trained (blue) and purely supervised (red) models, in Figure 4A, is identical. The optimization procedure is greatly influenced by the learning rate, which should be kept within a moderate range to enable stable convergence without overshooting from the minima and diverging. As shown in Figure 4A, the purely supervised models (for certain weight initializations) get stuck in poor local minima, thereby yielding high variance (or standard deviations) on classification error. In contrast, the pre-trained models mostly enter the appropriate convergence routes without being trapped in poor local minima consistently yielding lower error with increasing number of iterations. Among the supervised models that did not get stuck in bad local minima, the pre-trained models still outperform them in terms of classification performance. We conduct a similar comparison as that of Figure 4A for the deep network topology as illustrated in Figure 4B. We observe similar results with the pre-trained model (blue) yielding a lower classification error than a purely supervised model (red). In fact, the pre-trained model converges to a lower classification error with fewer number of iterations, which establishes the effectiveness of the STDP-based pre-training procedure. It is noteworthy to mention that deep networks (in case of purely supervised training) do not get stuck in poor local minima for different initializations due to the enriched parameter space available for optimization. This enriched parameter space also allows us to use a higher learning rate without overfitting. We observed that increasing the learning rate significantly lowers the classification error achieved with the pre-trained model (yellow in Figure 4B). Additionally, the classification error of pre-trained model shows lower variance than the purely supervised networks that started independently from different initialized weights as described in Table 2. Thus, we can infer that STDP-based pre-training improves the robustness of the overall learning procedure.

**Table 2:** Learning rate and mean standard deviation of classification errors in shallow and deep multi-layer networks.

**Network topology**	**Shallow multi-layer network**		**Deep multi-layer network**		
**Model (Corresponding**	**Without Pre-training**	**With Pre-training**	**Without Pre-training**	**With Pre-training**	**With Pre-training**
**Models in Figure 4)**	**(Red) (Blue)**	**(Red)**	**(Blue)**	**(Yellow)**
Learning Rate	0.4	0.4	0.18	0.18	0.35
Variance (Mean STD)	10.57%	0.083%	0.146%	0.110%	0.099%

**Figure 5:**
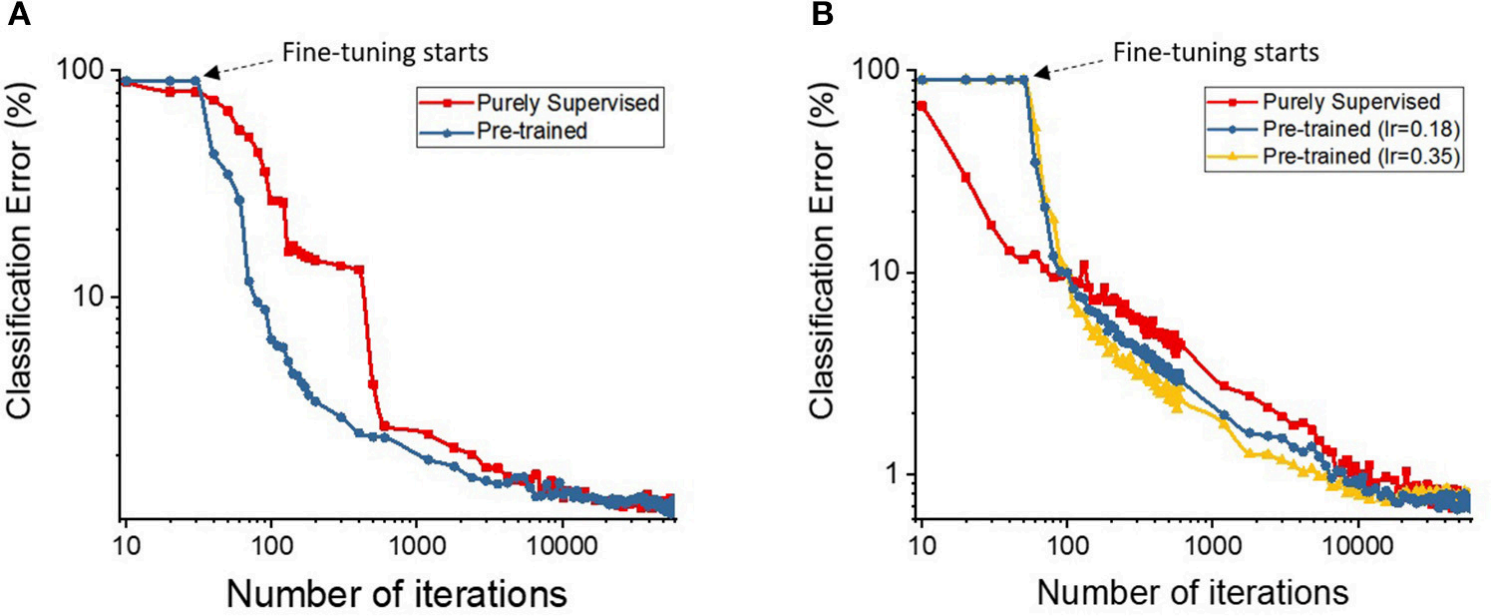
The plots show the classification accuracies on **(A)** shallow and **(B)** deep multi-layer convolutional spiking neural network as the semi-supervised optimization runs. The x-axis is the number of iterations (in log scale) and y-axis is classification accuracies (in log scale) on testing data.

**Figure 6:**
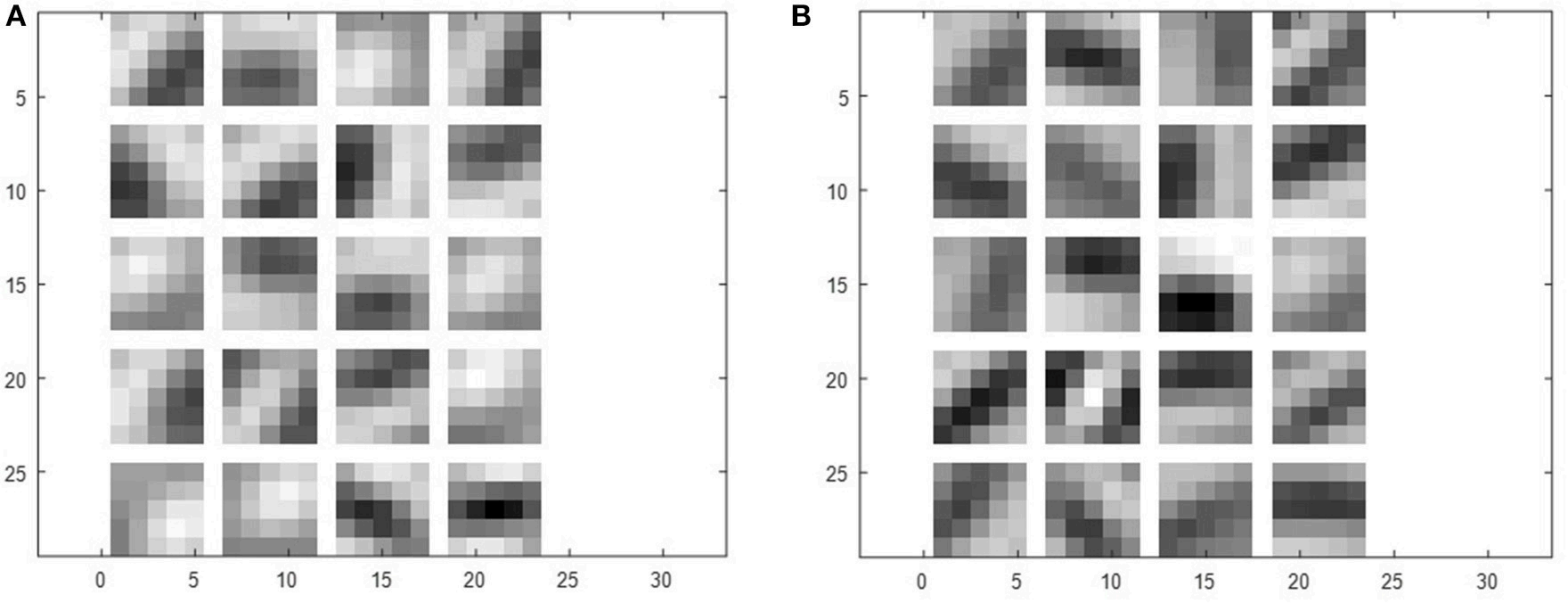
The weight kernels of **(A)** purely supervised and **(B)** pre-trained model in first convolutional layer.

To further quantify the benefits of the STDP-based pre-training method, we plotted the classification errors with respect to training efforts for both the purely supervised and pre-trained models that have identical weight initialization in the beginning of training. We quantify training effort as the total number of training iterations required for error convergence. The plots in Figure 5 illustrate the classification performance of the pre-trained model (blue, yellow) with respect to the purely supervised model (red). We observe that the pre-trained model (yielding very high error during the unsupervised pre-training stage) starts to outperform the purely supervised model with supervised fine-tuning yielding consistently lower error for both the shallow and deep topologies. Note, the classification error remains high initially (~90%) in case of a pre-trained model, because the fully-connected layers are not trained during the STDP-based pre-training phase. Besides lower error rate, the proposed semi-supervised training also yields faster training convergence. Specifically, the convergence time (in which the shallow multi-layer network reaches 2% classification error) with STDP-based pre-training (1,200 iterations) is significantly lower than that of purely supervised case (3,000 iterations). Similarly, the pre-trained deep network achieves 1% classification error after 4,800 iterations, whereas the randomly initialized network with spike-based BP takes 10,200 iterations. Essentially, the speed of optimization to reach certain amount of testing error improves by ~2.5 × for both shallow and deep multi-layer network with STDP pre-training as compared to purely supervised gradient BP. The boosted performance of gradient-based supervised fine-tuning provides an insight that the efficient unsupervised feature learning prior to the fine-tuning phase significantly reduces the training effort to facilitate convergence. We believe that unsupervised initialization helps to mitigate the difficult highly non-convex optimization problem by better initializing and conditioning the network parameters. Eventually, the classification accuracies of shallow multi-layer network saturates at the amount of lowest error rates of 1.20% (purely supervised model) and 1.23% (pre-trained model) averaged over 130–150 (78000–90000) training epochs (iterations). The classification errors of purely supervised model and pre-trained model for training deep multi-layer networks saturate at the 0.77 and 0.72%, respectively. The classification results shown are comparable to the state-of-the-art results as compared in Table 3. Figure 6 shows the adjusted weight kernels in first convolutional layer for purely supervised (A) and pre-trained (B) model after 150 training epochs. The weight kernels of the pre-trained model in Figure 6B indicate more definite shapes of pattern characteristics compared to those from the purely supervised model in Figure 6A.

**Table 3:** Comparison of the SNNs classification accuracies on MNIST digit recognition task.

**Model**	**Architecture**	**Learning method**	**Accuracy**
Esser et al., 2015	Deep Fully-connected	Offline learning, conversion	99.42%
Hunsberger and Eliasmith, 2015	Deep Fully-connected	Offline learning, conversion	98.37%
Diehl et al., 2016	Deep Convolutional	Offline learning, conversion	99.1%
Diehl and Cook, 2015	Two-layer Fully-connected	Unsupervised STDP	95.0%
Kheradpisheh et al., 2016	Deep Convolutional	Layerwise STDP + SVM classifier	98.4%
Panda and Roy, 2016	Deep Convolutional	Convolutional Autoencoder	99.05%
Lee et al., 2016	Deep Convolutional	Backpropagation	99.31%
Semi-supervised Learning (This work)	Deep Convolutional	STDP-based Pretraining + Backpropagation	99.28%

**Figure 7:**
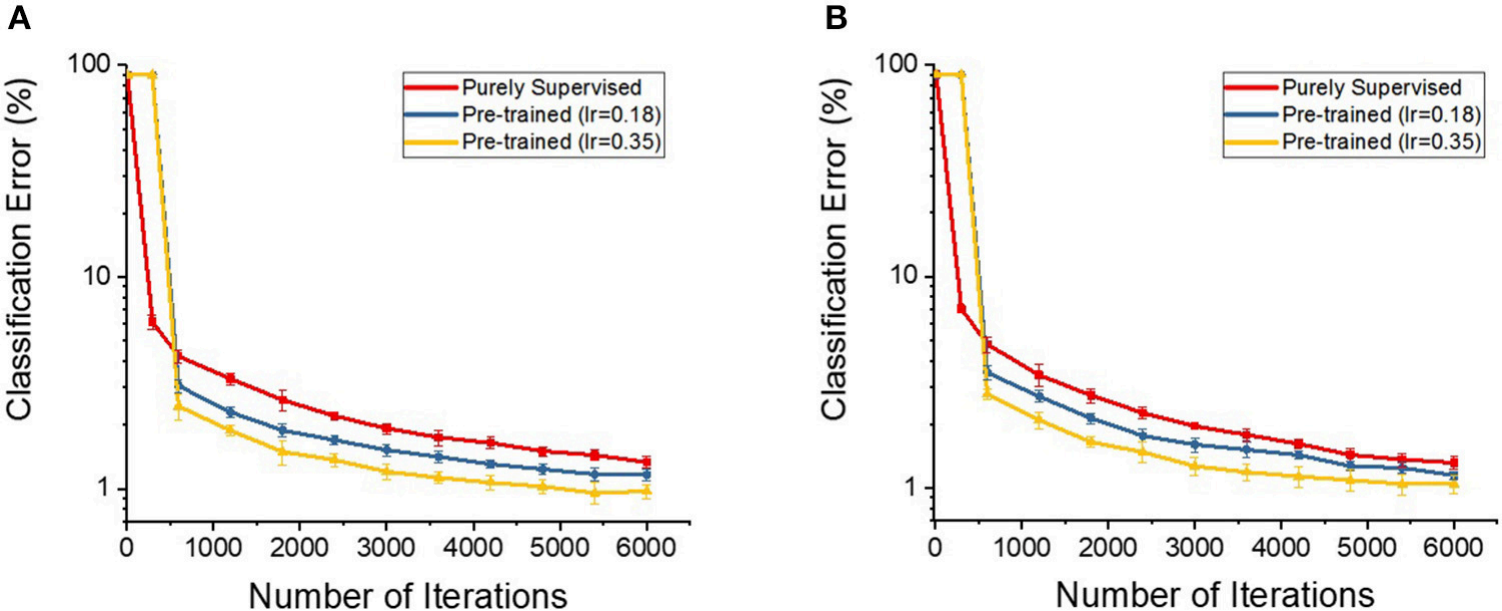
The classification accuracies (in log scale) on the deep multi-layer convolutional spiking neural networks of pre-trained and supervised model starting from **(A)** Lee initialization **(B)** Glorot initialization.

Lastly, lets try to answer the following question: Does the STDP-based pre-training also provide the benefits when the network is initialized with different random initialization? To address this question, we perform an experiment that initializes the parameters of deep multi-layer SNNs with another initialization scheme [“Glorot initialization”(Glorot and Bengio, 2010)] and train with the proposed semi-supervised learning strategy. We use unsupervised STDP to pre-train the SNNs (initialized with “Glorot initialization”) and measure the classification performances (that started from 10 different states of random weights) while fine-tuning the networks with gradient descent backpropagation algorithm. The classification performance shows faster training convergence (1,800 iterations to reach 2% error) and improved robustness compared to the networks without STDP-based pre-training (3,000 iterations to reach 2% error). Note that pre-trained models (initialized with “Glorot initialization”) show slightly slower training convergence time compared to Lee Initialization (Lee et al., 2016) pre-trained models (1,200 iterations to reach 2% error). Figure 7 shows the classification performances with respect to training efforts for the purely supervised and pre-trained models of each initialization scheme [(a) Lee initialization vs. (b) Glorot initialization]. Figure 7B and Table 4 depict similar trends: pre-trained models achieve better classification performances and lower variances (measured from 10 different states of random weights) compared to purely supervised models. Therefore, we infer that STDP-based pre-training also helps to better initialize and condition the network parameters in different initialization scheme such as “Glorot initialization.”.

**Table 4:** Mean standard deviation of classification errors that are initialized with different weight initialization schemes in deep multi-layer networks.

**Model (Corresponding models in Figure 7)**	**Without pre-training (Red)**	**With pre-training (Blue)**	**With pre-training (Yellow)**
(Lee et al., 2016) Initialization	0.146%	0.110%	0.099%
(Glorot and Bengio, 2010) initialization	0.171%	0.131%	0.116%

## 4. Discussion

Our proposal of STDP-based unsupervised pre-training is demonstrated to achieve improved robustness and significant speed-up in training procedure. Conceptually, the benefits of the semi-supervised learning strategy come from the inherent attributes of two different learning mechanisms. First, the unsupervised STDP learning automatically determines the useful features from high-dimensional input patterns that strengthens the connections between strongly correlated neurons. Hence, the quick and simple modifications are facilitated so that the non-linear representations are simply extracted based on the degree of correlation between neurons in adjacent layers. Moreover, the nature of unsupervised STDP learning is less prone to the overfitting problem than the supervised learning (Kheradpisheh et al., 2016). These peculiarities allow the unsupervised STDP mechanism to be an effective initializer for directing the network to an optimal starting point in the parameter space at the beginning of gradient descent optimization. On the other hand, supervised BP learning is a complex, global and gradient-based algorithm, which adjusts the synaptic weights proportional to the degree of their contributions to the final loss in the direction of minimizing the errors. The gradient descent algorithm is susceptible to the initial condition of network parameters, which causes variable convergence and necessitates large number of training data to generalize the network well. Note that there are numerous studies to appropriately initialize the network parameters in the domain of ANN (Erhan et al., 2009; Glorot and Bengio, 2010; He et al., 2015). In SNNs, the conversion from adapted ANN to SNNs (Cao et al., 2015; Hunsberger and Eliasmith, 2015; Diehl et al., 2016; Rueckauer et al., 2017; Sengupta et al., 2018) is one popular methodology to take advantage of state-of-the-art deep learning algorithms and techniques. The conversion technique shows remarkable classification performances, nevertheless there are issues that prevent them from becoming universal. It is inevitable to avoid the classification accuracy degradation due to ANN-to-SNN conversion, which becomes higher when dealing with real sensory data from event-driven dynamic vision sensors (Lichtsteiner et al., 2006; Delbrück et al., 2010). The weight-normalization scheme, which effectively converts the network parameters, is still an active research field. In addition, the privacy issues can not be overlooked in case of disclosing, sharing and destroying the personal (credential) data generated from edge devices for ANN training in cloud services (or data centers). Consequently, all-spiking neural network systems, which efficiently train and test the deep SNNs by direct input spike events, allow to protect privacy and increase the availability of private data to the artificial intelligence systems. As mentioned before, the initial conditions of SNN are pre-defined based on the network parameters, which are the synaptic weights and firing threshold of spiking neurons. However, it is still not evident how to initialize the multi-layer SNN systems in an optimal way. In this work, we leverage STDP unsupervised learning to appropriately initialize the network parameters in a data-driven manner prior to the supervised gradient descent BP learning.

**Figure 8:**
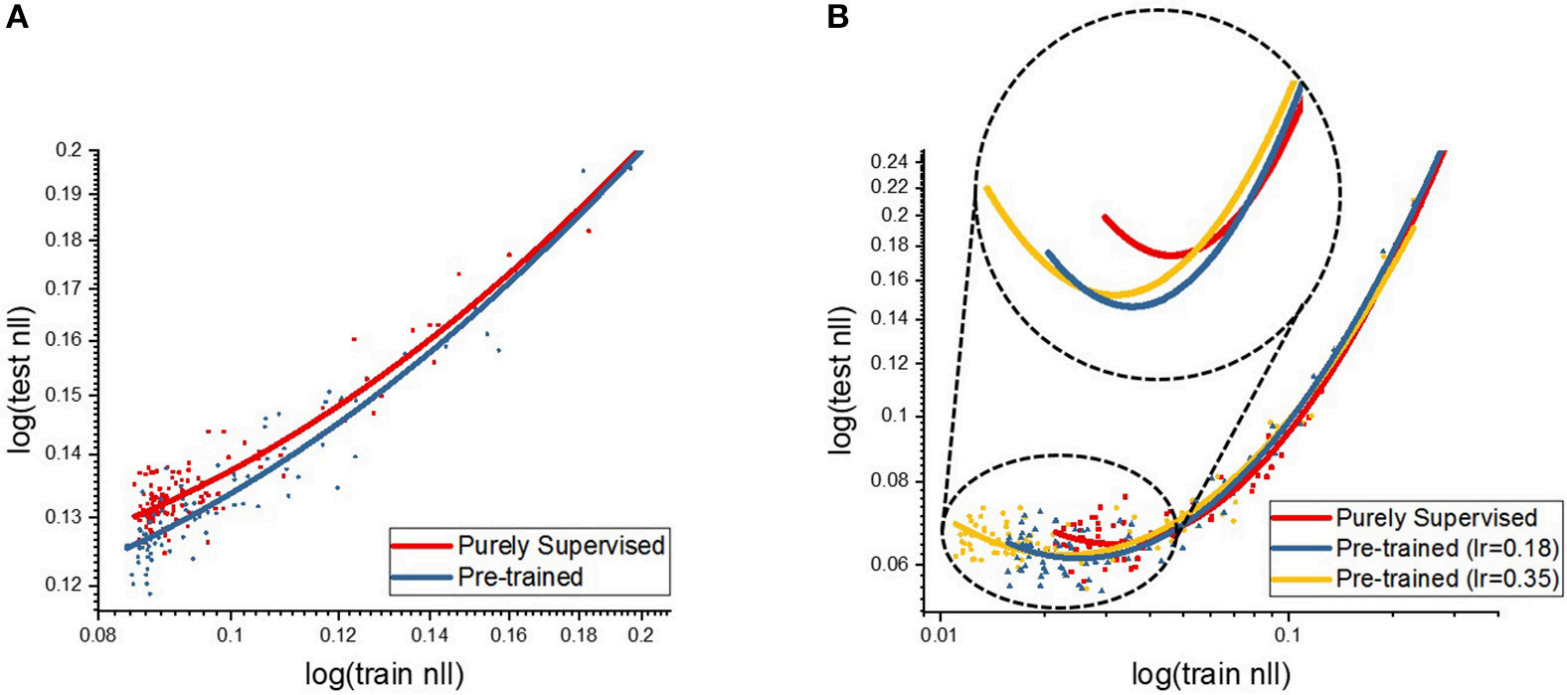
The plots show the NLL cost on **(A)** the shallow and **(B)** deep multi-layer convolutional spiking neural network. The horizontal and vertical axis indicate the NLL costs (in log scale) on training and testing data, respectively.

We performed an additional experiment to investigate how the proposed STDP-based unsupervised pre-training helps the subsequent gradient-based supervised fine-tuning compared to purely supervised training from random weight initialization. We hypothesize that unsupervised pre-training effect helps either optimize or generalize the systems. In this context, the optimization helps to locate the network to a better starting point in the parameter space, which induces lower training error. On the other hand, the generalization effect prevents the network from overfitting too closely to training sample, which results in lowering the errors on data that are not seen during the training. We trained shallow and deep multi-layer networks over 150 epochs with and without pre-training and evaluated the component sum of negative-log-likelihood (NLL) costs on testing and training data to highlight the performance gap between the two scenarios. The negative-log-likelihood function is formulated below.

(13) $$\begin{array}{r} NegativeLogLikelihood=\overset{n^{L}}{\underset{i=1}{\sum }}x_{i}\log p_{i}\left(x\right)+\left(1-x_{i}\right)\log\left(1-p_{i}\left(x\right)\right) \end{array}$$

where *n*^*L*^ represents the size of final layer, *x* is the output target labels and *p*(*x*) denotes the normalized firing rate of final output neurons. Figure 8 presents the testing NLL cost with respect to the training NLL cost for both shallow and deep network optimization. Table 5 shows the testing and training NLL costs averaged over 130–150 epochs. During the supervised BP learning, the pre-trained model yields a lower training NLL cost with the same training effort (representative of faster convergence) and the final training NLL cost of the pre-trained model saturates at a lower range than the purely supervised model as depicted in Table 5. This trend indicates that the unsupervised initialization induces the systems to be rapidly optimized and achieves better training error. The unsupervised pre-training, in effect, initially deploys the network to a parameter space where the initial point is closer to the local optima. In addition, we analyzed the test cost with respect to the training cost to measure the generalization effect of unsupervised pre-training. As the optimization proceeds toward the end, the testing NLL cost value saturates or starts to slightly increase because of overfitting, whereas the training NLL cost continually decreases as shown in Figure 8. However, we observe that the overfitting phenomenon occurs at the stage of lower training NLL cost in case of pre-trained models (for both shallow and deep cases) in comparison to the purely supervised training. The inset in Figure 8B highlights this effect wherein we observe that the pre-trained models saturates to lower convergence region (testing NLL cost) while delaying the overfitting phenomena. Note, overfitted neural network systems perform worse on test data (or data unseen during the training). Therefore, we infer that the pre-trained model can generalize better than the purely supervised model by means of pre-conditioning of the network parameters such that overfitting issue is mitigated. In essence, the STDP-based unsupervised initialization scheme provides an equivalent effect of classic regularization techniques such as early stopping (Caruana et al., 2001), *L*1/*L*2 weight decay (Hanson and Pratt, 1989) and dropout (Srivastava et al., 2014), which explicitly constrain the training model like adding penalty to the loss function or adding restriction on parameters.

**Table 5:** Final testing and training NLL costs (averaged out over 130–150 epochs) in shallow and deep multi-layer networks.

**Network topology**	**Shallow multi-layer network**		**Deep multi-layer network**		
Model (Corresponding	With Pre-training	With Pre-training	Without Pre-training	With Pre-training	Without Pre-training
Models in Figure 8)	(Red)	(Blue)	(Red)	(Blue)	(Yellow)
Final Testing NLL	0.1317	0.1266	0.0658	0.0627	0.0659
Final Training NLL	0.0894	0.0861	0.0234	0.0169	0.0118

## 5. Conclusion

Recent efforts in spiking neural networks have been focused toward building multi-layer systems to hierarchically represent highly nonlinear and complex functions. However, training hierarchical systems remains a difficult problem because of their inherent high dimensionality and non-convexity. In this work, we have shown that the convolutional spiking neural network comprising multiple hidden layers can be pre-trained with layer-wise unsupervised STDP learning and fine-tuned with supervised gradient descent BP algorithm. The unsupervised pre-training extracts the underlying structures from high dimensional input patterns in order to better initialize the parameters and supervised gradient-based BP algorithm takes the hierarchical system to optimal local minima. The proposed semi-supervised strategy benefits the training procedure to be more invariant to randomly assigned initial parameters, yields faster training and better generalization compared to purely supervised optimization without pre-training. We believe that STDP-based unsupervised initialization scheme coupled with state-of-the-art deep learning backpropagation algorithm can pave the way toward effectively optimizing deep spiking neural networks.

## Author contributions

CL and KR conceived the theory and research direction and CL implemented the algorithm and conducted the experiments. CL, PP, and GS discussed about the results and analysis, and wrote the manuscript.

### Conflict of interest statement

The authors declare that the research was conducted in the absence of any commercial or financial relationships that could be construed as a potential conflict of interest.
